# Comparison of ischemic preconditioning and BotulinumA Toxin injection for the prevention of ischemia-reperfusion injury in musculocutaneous flaps

**DOI:** 10.3906/sag-1904-95

**Published:** 2020-10-22

**Authors:** Handan DEREBAŞINLIOĞLU, Anıl DEMİRÖZ, Yağmur AYDIN, Hakan EKMEKÇİ, Özlem BALCI EKMEKÇİ, Övgü AYDIN, Levent CANKORKMAZ

**Affiliations:** 1 Department of Plastic Reconstructive and Aesthetic Surgery, Faculty of Medicine, Sivas Cumhuriyet University, Sivas Turkey; 2 Department of Plastic Reconstructive and Aesthetic Surgery, Faculty of Medicine, İstanbul University Cerrahpaşa Medical School, İstanbul Turkey; 3 Department of Biochemistry, Faculty of Medicine, İstanbul University Cerrahpaşa Medical School, İstanbul Turkey; 4 Department of Pathology, Faculty of Medicine, İstanbul University Cerrahpaşa Medical School, İstanbul Turkey; 5 Department of Pediatric Surgery, Faculty of Medicine, Sivas Cumhuriyet University, Sivas Turkey

**Keywords:** Ischemic preconditioning, Botulinum A toxin, ischemia-reperfusion injury, musculocutaneous flap, nitric oxide, myeloperoxidase

## Abstract

**Background/aim:**

The aim of the study was to evaluate the protective effect of Botulinum A toxin injection against ischemia-reperfusion injury.

**Materials and methods:**

Thirty-two Sprague-Dawley rats were divided into: control, ischemia-reperfusion, ischemic preconditioning, and botulinum groups. In all groups the musculocutaneous pedicle flap was occluded for 4 h, and then reperfused to induce ischemia-reperfusion injury. Serum and tissue myeloperoxidase (MPO) and nitric oxide (NO) levels were measured at 24 h and at 10 days.

**Results:**

Tissue MPO levels did not differ significantly between the ischemic preconditioning and botulinum groups at 24 h but was significantly lower in the botulinum group at 10 days. Tissue NO levels were significantly higher in the ischemic preconditioning group compared to the botulinum group at 24 h and at 10 days. Serum MPO showed no significant difference between these two groups at 24 h but was significantly lower in the ischemic preconditioning group compared to the botulinum group at 10 days. Serum NO levels were not significantly different at 24 h but significantly higher in the botulinum group at 10 days.

**Conclusion:**

Findings show that botulinum has a protective effect against the ischemia-reperfusion injury via increased NO and decreased MPO levels in tissue. Based on tissue NO levels, ischemic preconditioning was significantly higher than botulinum.

## 1. Introduction

Free or pedicled musculocutaneous flaps are used in the closure of many complicated defects. Despite advances in technology and surgical techniques, flap loss following reperfusion remains a serious problem due to ischemia-reperfusion injury (IRI) [1]. IRI occurs due to complex mechanisms including apoptosis, production of free oxygen radicals, platelet aggregation, complement system activation, and leukocyte-endothelial interaction. Surgical delay is the only realistic method clinically shown to increase flap survival. This method causes vascular dilation and increases blood flow in muscle and adjacent skin tissue. Ischemic preconditioning is a surgical delay technique that involves subjecting tissue to a short, nonlethal period of ischemia, which increases its resistance to damage due to subsequent episodes of ischemia. In addition to surgical delay, many pharmacological agents are now used to prevent IRI. 

Botulinum toxin is the strongest known paralytic agent. Botulinum A toxin has become widely used in the treatment of conditions associated with muscle overaction. In addition to its paralytic effect, Botulinum A toxin also influences muscle metabolism. Matic et al. demonstrated that Botulinum A toxin exerts effects similar to ischemic preconditioning on muscle tissue [2].

Therefore, this study was conducted to evaluate the effectiveness of Botulinum A toxin injection as a preconditioning technique to prevent IRI in rat musculocutaneous flaps.

## 2. Materials and methods

### 2.1. Subjects

The present study was performed in the Experimental Animals Laboratory of İstanbul University, Cerrahpaşa Medical School after receiving ethical approval from the İstanbul University Local Ethics Committee. Thirty-two female Sprague-Dawley rats with an average weight of 210 g (180–230 g) which were raised in the İstanbul University Experimental Medicine Research Center were used in the study. The rats were randomly separated into four groups: control group, ischemia-reperfusion (IR) group, ischemic preconditioning group, and the Botulinum A toxin group (Table 1).

**Table 1:** Groups and procedures.

The Groups	Procedures
Control group	No procedure was applied
Ischemia Reperfusion group	Ischemia reperfusion procedure
Ischemic preconditioning group	Ischemic preconditioning procedure + Ischemia reperfusion procedure
Botulinum A group	Botulinum A toxin injection + Ischemia reperfusion procedure

## 2.2. Surgical procedure

A dorsal paraspinal incision was made to expose the medial edges of the latissimus dorsi muscles. The flap of muscle with a 4 × 5 cm skin island was raised on a thoracodorsal pedicle. To prevent detachment of skin from the muscle, suspension sutures were placed in the inferior and superior corners of the flap’s medial edge. The skin island was completely circumcised to ensure supply from the muscular perfusing vessels. While raising the latissimus dorsi musculocutaneous flap (LDMF), all intercostal arteries feeding the muscle were ligated to ensure the flap was perfused exclusively through the thoracodorsal pedicle. Without fully skeletonizing the thoracodorsal pedicle, the surrounding muscle tissue was thinned enough to allow clamping.

## 2.3. Ischemic preconditioning procedure

The ischemic preconditioning method used in this study was based on the model described by Pang et al., in which they demonstrated the effect of ischemic preconditioning in porcine latissimus dorsi muscle by implementing 3 cycles of 10 min of ischemia followed by 10 min of reperfusion [3]. Ten days after ischemic preconditioning, the flaps were subjected to the IR procedure.

## 2.4. Botulinum A toxin injection

Botulinum A toxin (BOTOX ®Ireland) was delivered as an intramuscular injection (1.4 units) to the right latissimus dorsi muscle of 8 rats 4 weeks before the IR procedure. This is the dose used by Çelik et al. [4].

## 2.5. Ischemia reperfusion procedure

Ischemia was achieved by clamping the pedicle of the raised thoracodorsal pedicled LDMF. At the end of 4 h, the clamp was removed to allow reperfusion.

## 2.6. Biochemical analyses

Serum and tissue samples obtained from the rats were analyzed for nitric oxide (NO) and myeloperoxidase (MPO) levels. Blood samples (5 cc) were collected from 4 rats in each group at 24 h and 10 days after reperfusion of the LDMF. The blood samples were placed in dry tubes and centrifuged for 4 min at 4000 rpm. The serum samples were placed in polypropylene tubes and stored in liquid nitrogen.

Tissue specimens were obtained by removing the entire flap from the pedicle before decapitation of the rat. Biopsy materials were divided into two groups vertically and half of them were used for histopathological examination and the other group was used for tissue MPO and NO analysis.

The tissue was wrapped in aluminum foil and frozen in liquid nitrogen. The tissue specimens were cut with a Heidolph electric knife into 0.2 g pieces and homogenized for 5 min in 1 mL of phosphate buffer solution (pH 7.2). The homogenized tissues were centrifuged for 10 min at 5000 rpm in polypropylene tubes in a Sigma 3U18K centrifuge (Germany). The resulting supernatants were separated and used in analyses.

Tissue and serum NO levels were measured by colorimetric method using Biotech Nitric Oxide Assay Kit (Oxis Research, catalogue no: 22110, USA). Tissue and serum MPO levels were measured using the ELISA method using HK105 Rat MPO ELISA Kit (Hycult Biotech,Netherlands).

## 2.7. Histopathological analysis

**Figure 1:**
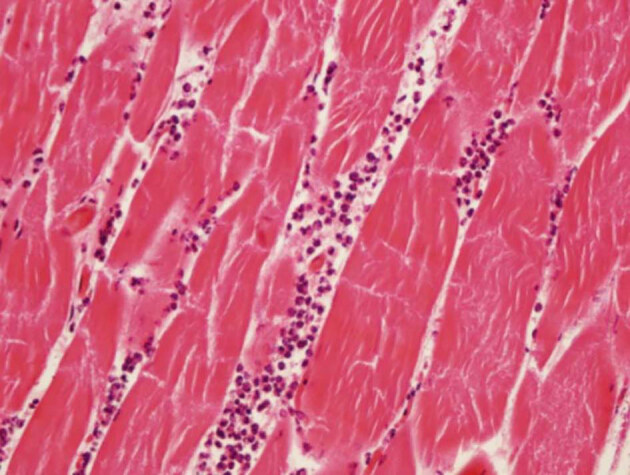
Grade 1 acute inflammation in muscle tissue.

**Figure 2:**
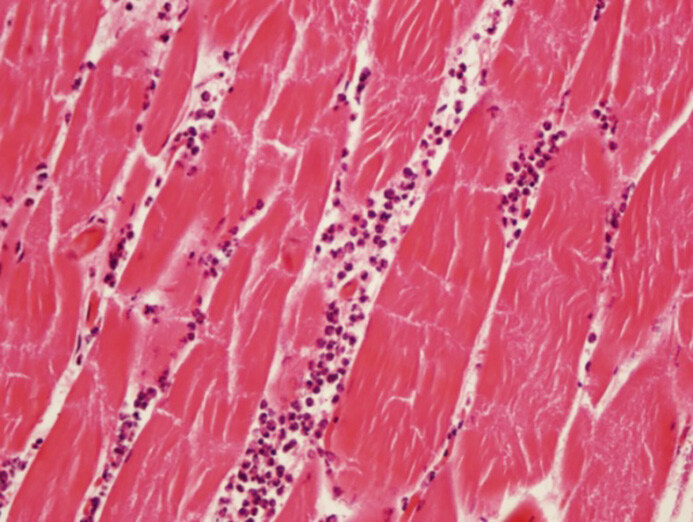
Grade 2 acute inflammation in muscle tissue.

**Figure 3:**
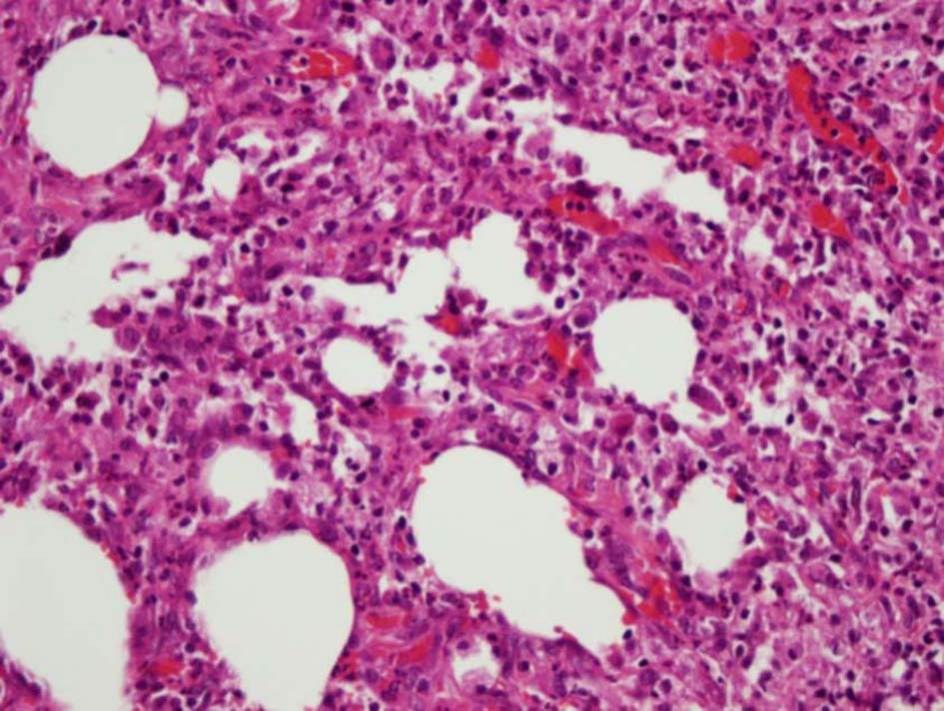
Grade 3 acute inflammation in muscle tissue.

Tissue samples were collected at the 24th hour and on the 10th day, before the rats were sacrificed. Tissue samples were formalin-fixed and embedded in paraffin after routine tissue follow-up and 4-µm sections were taken. The tissue samples were stained with hematoxylin eosin. The presence of acute inflammatory cells and the proliferation of vessels were examined in the dermis, the epidermis, the subcutaneous tissue and the muscle tissue with a light microscope; and also degeneration was evaluated in the muscle tissue. The histopathological above parameters were evaluated by a single pathologist and scored between 1 and 3 (Figures 1–3). The data obtained according to this scoring were evaluated statistically using the Kruskall–Wallis test, the Mann–Whitney U test, and the Wilcoxon signed rank test.

## 2.8. Groups 

### 2.8.1. Control group

No treatment was performed on 8 rats in the control group. The right latissimus dorsi muscles of these rats were raised as a musculocutaneous flap and they were cut off from the pedicle for tissue biopsy evaluation.

Serum MPO and NO levels were measured by applying the procedures described above to the blood samples taken from each rat. The values ​​obtained were used as base values.

## 2.8.2. Ischemia reperfusion group

The right latissimusdorsi muscle of 8 rats in the ischemia reperfusion group which were raised as musculocutaneous flaps were returned to their places after they were subjected to ischemia reperfusion procedures described above. MPO and NO levels were measured in blood and tissue samples of 4 rats at the 24th hour and 4 rats on the 10th day of ischemia reperfusion. Thus, the effect of ischemia reperfusion injury on the parameters used was determined. The data of the other two experimental groups were compared with the data of this group and their protective effects were evaluated.

## 2.8.3. Ischemic preconditioning group

The right latissimus dorsi musculocutaneous flap of 8 rats in this group was raised and after ischemic preconditioning as described above was applied flaps were returned to their places. Ten days after the preconditioning flaps were raised again to be subjected to ischemia reperfusion procedure and were settled in their original places. MPO and NO levels were measured in blood and tissue samples of 4 rats at the 24th hour and 4 rats on the 10th day of ischemia reperfusion. Values ​​were compared with other groups.

## 2.8.4. Botulinum A toxin group

An intramuscular injection of botulinum A toxin was administered to the right latissimus dorsi muscle of 8 rats in this group. Four weeks after the application, the right latissimus dorsi musculocutaneous flap was raised in order to perform the IR procedure and the flaps were returned to their places. MPO and NO levels were measured in blood and tissue samples of 4 rats at the 24th hour and 4 rats on the 10th day of IR. Values ​​were compared with other groups.

## 2.9. Statistical analysis

Number Cruncher Statistical System 2007 & Power Analysis and Sample Size 2008 Statistical Software (Utah, USA) program was used for the statistical analyses of biochemical values. In addition to descriptive statistical methods (mean, standard deviation, and median), the Kruskal–Wallis test was used in comparisons between groups because the variables did not meet normal distribution criteria due to the number of subjects in each group. The Mann–Whitney U test was used to determine the group responsible for the difference. Bonferroni correction after multiple comparisons was not made in this study. Significance was evaluated at the level of P < 0.05.

## 3. Results

### 3.1. Tissue myeloperoxidase levels

Mean tissue MPO levels at 24 h were 0.38 ± 0.23 µmol/g wet tissue in the control group, 1.36 ± 0.12 µmol/g in the IR group, 1.06 ± 0.07 µmol/g in the preconditioning group, and 1.13 ± 0.03 µmol/g in the Botulinum A toxin group. There was a statistically significant difference among the groups (P < 0.01). MPO levels were significantly higher in the IR, preconditioning, and Botulinum A toxin groups when compared to the control group (P < 0.01) and in the IR group compared to the preconditioning and Botulinum A toxin groups (P < 0.01). MPO levels in the preconditioning and Botulinum A toxin groups did not differ significantly (P > 0.05).

At 10 days, mean tissue MPO levels were 0.38 ± 0.23 µmol/g wet tissue in the control group, 1.15 ± 0.06 µmol/g in the IR group, 1.12 ± 0.07 µmol/g in the preconditioning group, and 0.98 ± 0.09 µmol/g in the Botulinum A toxin group (P < 0.01). Mean MPO levels at 10 days were significantly higher in the IR, preconditioning, and Botulinum A toxin groups compared to the control group (P < 0.01). No significant difference was found between day 10 tissue MPO levels in the IR and preconditioning groups (P > 0.05), while the Botulinum A toxin group showed significantly lower levels compared to the IR group (P < 0.05). Tissue MPO levels were significantly higher in the preconditioning group than the Botulinum A toxin group (P < 0.05).

The IR group showed a significant decrease in tissue MPO levels at day 10 compared to 24 h (P < 0.05). The difference between the tissue MPO levels at 24 h and 10 days was not significant in the preconditioning and Botulinum A toxin groups (P > 0.05) (Table 2).

**Table 2:** Tissue myeloperoxidase measurements.

(microgram\gramwettissue)		Myeloperoxidase Tissue levels24th hour	Myeloperoxidasetissue levels10th day	aP
Control	Avarege	0.38	0.38	-
	SD	0.23	0.23	
	Median	0.29	0.29	
IR	Avarege	1.36	1.15	0.043*
	SD	0.12	0.06	
	Median	1.36	1.17	
Delay	Avarege	1.06	1.12	0.279
	SD	0.07	0.07	
	Median	1.04	1.15	
Botox	Avarege	1.13	0.98	0.068
	SD	0.03	0.09	
	Median	1.13	0.98	
bP		0.001**	0.001**	
cControl-IR		0.004**	0.004**	
cControl-Delay		0.004**	0.004**	
cControl-Botox		0.004**	0.004**	
cIR-Delay		0.009**	0.295	
cIR-Botox		0.009**	0.016*	
cDelay-Botox		0.095	0.032*	

aWilcoxon signed rank test bKruskal–Wallis test cMann–Whitney U test *P < 0.05 **P < 0.01

## 3.2. Tissue nitric oxide levels

Mean tissue NO levels at 24 h were 0.68 ± 0.24 µmol/g wet tissue in the control group, 0.88 ± 0.17 µmol/g in the IR group, 1.97 ± 0.26µmol/g in the preconditioning group, and 1.62 ± 0.10 µmol/g in the Botulinum A toxin group (P < 0.01). While no significant difference was detected between the control and IR groups (P > 0.05), tissue NO levels were significantly higher in the preconditioning and Botulinum A toxin groups when compared to the control group (P < 0.01) and the IR group (P < 0.01). Tissue NO level at 24 h was also significantly higher in the preconditioning group compared to the Botulinum A toxin group (P < 0.05).

At 10 days, mean tissue NO levels were 0.68 ± 0.24 µmol/g in the control group, 1.6 ± 0.14 µmol/g in the IR group, 1.97 ± 0.04 µmol/g in the preconditioning group, and 0.81 ± 0.31 µmol/g in the Botulinum A toxin group (P < 0.01). The IR and preconditioning groups showed significantly higher levels of tissue NO on day 10 compared to the control group (P < 0.01), whereas the difference between the Botulinum A toxin and control groups was nonsignificant (P > 0.05). Mean day 10 tissue NO level in the IR group was significantly lower than in the preconditioning group and significantly higher than in the Botulinum A toxin group (P < 0.01). Mean NO level was also significantly higher in the preconditioning group compared to the Botulinum A toxin group (P < 0.01).

The IR group showed a significant increase in tissue NO levels between 24 h and 10 days (P < 0.05). A statistically significant change was not seen in the preconditioning group between 24 h and 10 days (P > 0.05). The Botulinum A toxin group showed a significant decrease in tissue NO levels between 24 h and 10 days (P < 0.05) (Table 3).

**Table 3:** Tissue nitric oxide measurements.

(micromole\gramwettissue)		Nitric oxidetissue levels24th hour	Nitric oxideTissue levels10th day	aP
Control	Average	0.63	0.63	-
	SD	0.24	0.24	
	Median	0.55	0.55	
IR	Average	0.88	1.63	0.043*
	SD	0.17	0.14	
	Median	0.90	1.60	
Delay	Average	1.97	1.97	0.893
	SD	0.26	0.04	
	Median	1.97	1.99	
Botox	Average	1.62	0.81	0.043*
	SD	0.10	0.31	
	Median	1.62	0.81	
bP		0.001**	0.001**	
cControl-IR		0.104	0.004**	
cControl-Delay		0.004**	0.004**	
cControl-Botox		0.003**	0.372	
cIR-Delay		0.009**	0.009**	
cIR-Botox		0.009**	0.009**	
cDelay-Botox		0.036*	0.009**	

aWilcoxon signed rank test bKruskal–Wallis test cMann–Whitney U test *P < 0.05 **P < 0.01

## 3.3. Serum myeloperoxidase levels

Mean serum MPO levels at 24 h were 319.77 ± 7.52 ng/mL in the control group, 416.42 ± 15.75 ng/mL in the IR group, 466.42 ± 15.75 ng/mL in the preconditioning group, and 470.46 ± 5.65 ng/mL in the Botulinum A toxin group (P < 0.01). Serum MPO levels were significantly higher in the IR, preconditioning, and Botulinum A toxin groups compared to the control group (P < 0.01) and in the preconditioning and Botulinum A toxin groups compared to the IR group (P < 0.01). No significant difference in 24 h serum MPO was detected between the preconditioning and Botulinum A toxin groups (P > 0.05).

At 10 days, mean serum MPO levels were 319.77 ± 7.52 ng/mL in the control group, 451.62 ± 14.10 ng/mL in the IR group, 441.02 ± 1.62 ng/mL in the preconditioning group, and 457.14 ± 10.98 ng/mL in the Botulinum A toxin group (P < 0.01). Mean day 10 serum MPO levels were significantly higher in the IR, preconditioning, and Botulinum A toxin groups compared to the control group (P < 0.01). Although the differences between the IR group and the preconditioning and Botulinum A toxin groups did not reach statistical significance (P > 0.05), serum MPO levels on day 10 were significantly lower in the preconditioning group when compared with the Botulinum A toxin group (P < 0.05).

In the preconditioning group, serum MPO was significantly lower at 10 days compared to values at 24 h (P < 0.05). In the IR and Botulinum A toxin groups, no statistically significant change in serum MPO occurred between 24 h and 10 days (P > 0.05) (Table 4).

**Table 4:** Serum myeloperoxidase measurements.

(nanogram\milliliter)		Myeloperoxidase Serum levels24th hour	Myeloperoxidase Serum levels10th day	aP
Control	Average	319.77	319.77	-
	SD	7.52	7.52	
	Median	319.40	319.40	
IR	Average	416.42	451.62	0.080
	SD	15.75	14.10	
	Median	416.42	454.00	
Delay	Avarege	466.42	441.02	0.043*
	SD	9.78	1.62	
	Median	466.80	440.30	
Botox	Average	470.46	457.14	0.080
	SD	5.65	10.98	
	Median	470.80	458.80	
bP		0.001**	0.002**	
cControl-IR		0.006**	0.006**	
cControl-Delay		0.006**	0.006**	
cControl-Botox		0.006**	0.006**	
cIR-Delay		0.009**	0.117	
cIR-Botox		0.009**	0.402	
cDelay-Botox		0.295	0.047*	

aWilcoxon signed rank test bKruskal–Wallis test cMann–Whitney U test *P < 0.05 **P < 0.01

## 3.4. Serum nitric oxide levels

Mean serum NO levels at 24 h were 17.56 ± 4.22 µmol/L in the control group, 15.36 ± 3.02 µmol/L in the IR group, 42.70 ± 3.58 µmol/L in the preconditioning group, and 46.52 ± 5.90 µmol/L in the Botulinum A toxin group (P < 0.01). There was no significant difference between the control and IR groups (P > 0.05), while serum NO levels were significantly higher in the preconditioning and Botulinum A toxin groups compared to the control group (P < 0.01). Accordingly, serum NO levels were significantly higher in the preconditioning and Botulinum A toxin groups when compared to the IR group (P < 0.01). However, 24 h serum NO levels did not differ significantly between the preconditioning and Botulinum A toxin groups (P > 0.05).

On day 10, mean serum NO levels were 17.56 ± 4.22 µmol/L in the control group, 22 ± 3.67 µmol/L in the IR group, 25.09 ± 3.04 µmol/L in the preconditioning group, and 35.84 ± 2.59 µmol/L in the Botulinum A toxin group (P < 0.01). While no statistical significant difference was detected between the control and IR groups (P > 0.05), both the preconditioning and Botulinum A toxin groups had significantly higher levels when compared with the control group (P < 0.01). No significant difference in day 10 serum NO levels was detected between the IR and preconditioning group (P > 0.05), whereas the IR group had significantly lower levels than the Botulinum A toxin group (P < 0.01) and the preconditioning group had significantly lower levels compared to the Botulinum A toxin group (P < 0.01).

The IR group showed a significant increase in serum NO levels at 10 days compared to 24 h (P < 0.05), while the preconditioning and Botulinum A toxin groups exhibited significant decreases (P < 0.05) (Table 5).

**Table 5:** Serum nitric oxide measurements.

(micromole\liter)		Nitric oxideserum levels24th hour	Nitric oxideserum levels10th day	P
Control	Average	17.56	17.56	-
	SD	4.22	4.22	
	Median	18.80	18.80	
IR	Average	15.36	22.00	0.043*
	SD	3.02	3.67	
	Median	15.50	22.00	
Delay	Average	42.70	25.09	0.043*
	SD	3.58	3.04	
	Median	42.30	25.50	
Botox	Average	46.52	35.84	0.043*
	SD	5.89	2.59	
	Median	46.70	36.70	
bP		0.001**	0.001**	
cControl-IR		0.255	0.123	
cControl-Delay		0.004**	0.007**	
cControl-Botox		0.004**	0.004**	
cIR-Delay		0.009**	0.117	
cIR-Botox		0.009**	0.009**	
cDelay-Botox		0.347	0.009**	

aWilcoxon signed rank test bKruskal–Wallis test cMann–Whitney U test *P < 0.05 **P < 0.01

## 3.5. Histopathological result 

**Figure 4:**
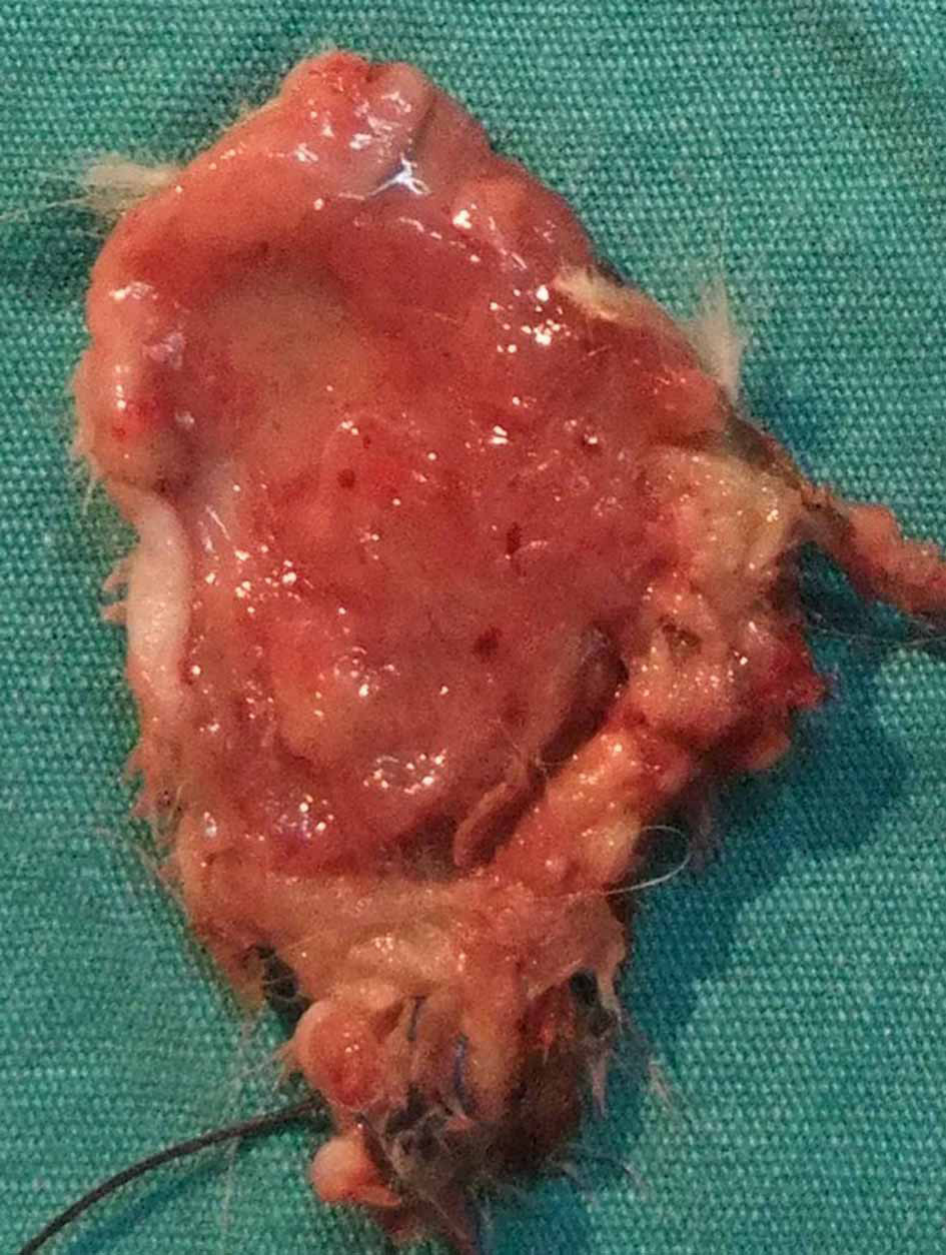
Rat pedicled latissimus dorsi musculocutaneous flap 10 days after exposure to ischemia-reperfusion (silk-marked tip distal margin).

**Figure 5:**
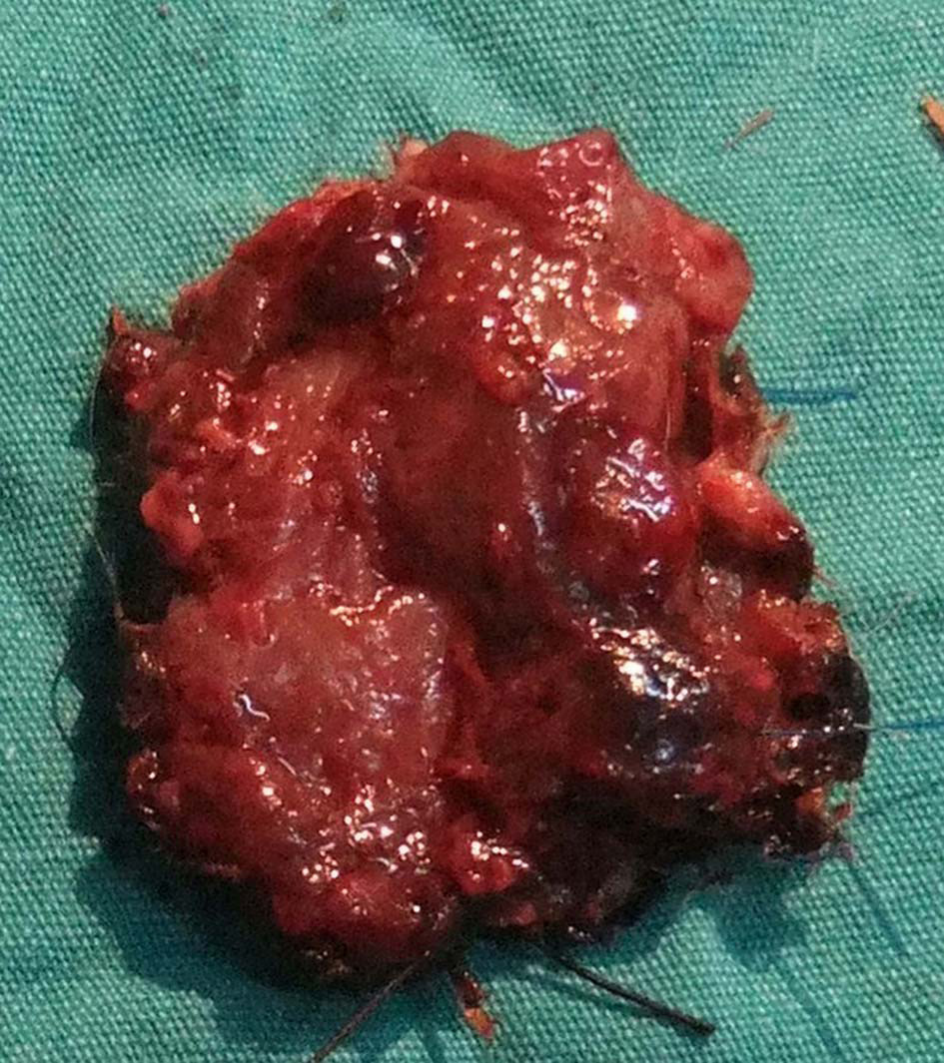
Rat pedicled latissimus dorsi musculocutaneous flap 10 days after ischemic preconditioning followed by ischemiareperfusion (silk-marked tip distal margin).

**Figure 6:**
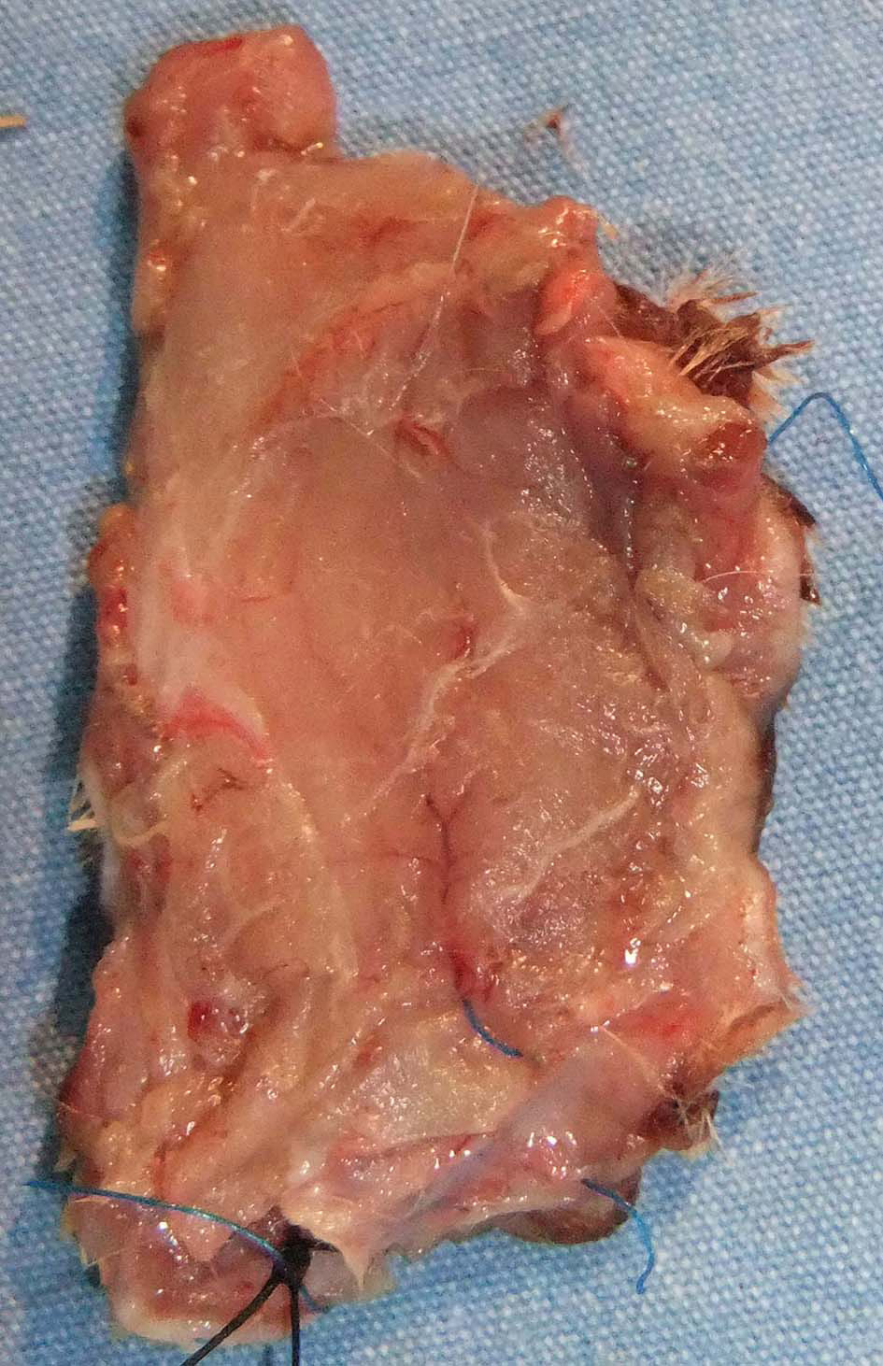
Rat pedicled latissimus dorsi musculocutaneous flap 10 days after Botulinum A injection followed by ischemiareperfusion (silk-marked tip distal margin).

In the group subjected exclusively to ischemia-reperfusion, the distal portion of the flap exhibited minimal necrosis and loss of muscle tissue under macroscopic examination as well as microscopic ulceration and necrosis in the epidermis on day 10. Macroscopic or microscopic necrosis was not observed in the other groups (Figures 4–6). According to the histopathological examination, acute inflammation of the Botulinum A toxin group of dermis tissue was more severe than the IR group at the 24th hour and on the 10th day. The acute inflammation at the 24th hour and on the 10th day in the Botulinum A toxin group of muscle tissue was less than the IR group, but more severe than the preconditioning group.

In the biopsy taken from the Botulinum A toxin group at 24 h, more vascularization was observed in the dermis layer compared to the control group. The vascularization of the muscle tissue was similar to the control group at the 24th hour, but vascularity in the Botulinum A toxin group was higher than the control group on the 10th day.

On the 10th day, muscle degeneration in the preconditioning group was higher than the IR and the Botulinum A toxin groups.

## 4. Discussion

With the discovery that Botulinum A toxin inhibited acetylcholine release in motor neurons, Botulinum A toxin began to be used in the treatment of many diseases. When the molecular structure and mechanism of action of the toxin was later understood, experimental studies demonstrated that the toxin affects not only motor neurons but also the autonomous nervous system. Sympathetic stimulation of vessels initially causes vasodilatation due to the effect of NE on β1 receptors and later vasoconstriction due to α2 receptor activation. Botulinum A toxin has been shown to reduce NE-mediated vasoconstriction [5]. Perivascular administration of Botulinum A toxin increases vessel diameter and tissue circulation [2,6]. This suggests the possible utility of botulinum toxin in flap surgery to increase flap survival. Botulinum A toxin was found to increase flap survival in subcutaneous applications [7,8].

Preconditioning has been shown in multiple studies to clinically increase flap survival [9,10]. This technique provides late phase protection against skeletal muscle necrosis and requires multiple IR cycles [11]. However, disadvantages of this method include the need for an additional surgical session to perform the preconditioning procedure and the subsequent increases in length of hospital stay, infection risk, and cost.

Matic et al. investigated the effects of Botulinum A toxin on muscle metabolism in rabbit masseter muscle. They reported increases in blood flow, blood volume, permeability surface area product, and glucose uptake in the muscle following botulinum toxin injection [2]. Clinical studies showed that surgical delay procedure increased the blood flow in muscle and adjacent skin tissue [12]. Although tissue subjected to ischemic preconditioning alters its energy metabolism to decrease energy need [3,13], glucose uptake in muscle tissue increases after botulinum injection [2]. These phenomena are contradictory.

In our study, macroscopic tissue necrosis was not observed in the Botulinum A toxin or the ischemic preconditioning group. In the group subjected exclusively to IR, the distal portion of the flap exhibited minimal necrosis and loss of muscle tissue on macroscopic examination, as well as microscopic ulceration and necrosis in the epidermis on day 10. In composite flaps, it has been shown that extended ischemia can cause muscle necrosis with no loss of skin vitality [14].

Eun et al. evaluated the protective effect of erythropoietin against IRI in musculocutaneous flaps. At 24 h, they detected lower MPO levels and higher NO levels in the erythropoietin-treated group compared to the group subjected to ischemia-reperfusion only. MPO level was evaluated as an indicator of neutrophil infiltration, and the authors concluded that erythropoietin protected against IRI by reducing inflammation and neutrophil infiltration and increasing tissue NO levels [15]. In the present study, we also considered elevated NO levels detected with colorimetric method and reduced MPO levels detected with ELISA method as a protective mechanism against IRI.

Endothelium produces NO, a well-known protective mediator against IRI [14]. NO is synthesized from the guanidinium group of L-arginine by NO synthetases (NOS). NO is an important chemical agent with various biological functions such as endothelium-dependent vasodilatation, clearing oxygen radicals, inhibiting platelet aggregation, and reducing leukocyte-endothelial adhesion [16]. Researchers have observed that while the use of NO synthetases inhibitor increased flap necrosis, L-arginine increased flap vitality [17]. 

In our study, tissue and serum NO levels were measured at 24 h and 10 days after IR. NO levels at 24 h were significantly higher in the groups that underwent ischemic preconditioning and Botulinum A toxin injection, with a greater increase in the preconditioning group. This suggests that while the protective effect of NO against IRI was present in both groups, ischemic preconditioning was superior to Botulinum A toxin. Although the IR-only group showed a statistically significant increase in tissue NO values on day 10, this increase was also observed in the serum values.

Ischemic preconditioning is evaluated in two periods, early protection (first window) and late protection (second window), and NO is involved in both. The effects of early preconditioning emerge within minutes of reperfusion and last for 2–3 h. The effects of late preconditioning become evident 12–24 h after reperfusion and last 2–3 days [18]. Xuan et al. showed that in tissue subjected to preconditioning, the postischemia release of NO during the first hours is based on endothelial NOS, while the NO released during the second window was mainly produced by late activation of macrophage-inducible NOS [19]. Other authors argued that endothelial NOS acts as a trigger and inducible NOS as a late-stage mediator [20]. According to our findings, the change in tissue NO levels between hour 24 and day 10 in the preconditioning group was statistically insignificant. This suggests that the protective effect of the preconditioning method seen at hour 24 was fully maintained on day 10.

Increased inducible NOS (iNOS) level following cutaneous Botulinum A toxin injection has been demonstrated by polymerase chain reaction (PCR) [21]. It was also shown that Botulinum A toxin increases survival of rat dorsal skin flaps [7]. In another study, histological examination revealed an increase in vessel diameter and neovascularization following cutaneous Botulinum A toxin injection. PCR was also used to show that vascular endothelial growth factor, which causes endothelial proliferation, was increased following Botulinum A toxin injection, along with vasodilatation, CD 31 (PECAM1 platelet-endothelial cell adhesion molecule) and iNOS [21]. The high tissue NO levels measured at 24 h in the Botulinum A toxin group compared to the control and IR groups may show the protective effect of the Botulinum A toxin against IRI by increasing NO levels.

In the literature, it has been proposed that the functional cut-off from the target organ that occurs during temporary muscle paralysis following intramuscular Botulinum A toxin injection may stimulate NO synthesis in motor neurons to preserve axonal vitality and ensure cell survival, resulting in a local increase in NO [22]. In complete ischemia, nerve function rapidly deteriorates within 30 to 90 min [23]. The degree of neural tissue damage occurring during ischemia is related to ischemia duration and blood flow during this period [24]. In our study, there was a significant decrease in tissue NO levels in the Botulinum A toxin group between 24 h and 10 days. This may be attributed to reduced neuronal release of NO due to IRI. These findings indicate that the NO-mediated protective effect of Botulinum A toxin decreased at 10 days. However, serum NO values in the Botulinum A toxin group were substantially higher the day 10 compared to the other groups. This shows that vessel proliferation increases NO levels in the blood. However, a problem preventing the movement of NO into the tissue could explain the reduced local effect of Botulinum A toxin. Moreover, considering that MPO activity leads to NO consumption [25], the high serum MPO levels in the Botulinum A toxin group on day 10 suggest that MPO may have inactivated NO before it could act in the tissue. This may mean that the oxidant-antioxidant equilibrium is restored within 10 days of IR and this is another reason for NO reduction. 

Myeloperoxidase is the green hemoprotein enzyme found in the azurophilic granules of polymorphonuclear leukocytes (PML). MPO enzymes turn H2O2 to HClO­ (hypochlorous radical) in the presence of Cl­. HClO­ is a strong oxidant agent, and kills the cell via protein and lipid peroxidation [26]. MPO measurement is used to calculate PML content and to measure inflammation in tissues such as the skin, eye, liver, and intestine [25]. MPO activity in the skin is a marker of neutrophil infiltration of the flap [13]. High MPO activity indicates NO consumption and the onset of endothelial dysfunction [27]. In our study, tissue and serum MPO levels measured at 24 h and 10 days after IR showed significantly lower tissue MPO levels in the control group compared to each of the other three IR groups. This shows the presence of neutrophil-mediated tissue damage in all of the three ischemia reperfusion groups. In statistical analyses, the Botulinum A toxin and preconditioning groups had significantly lower tissue MPO levels when compared with the ischemia-reperfusion group. This indicates lower levels of neutrophil-mediated tissue damage in the treated groups. Serum MPO is believed to be released into the vascular lumen by activated leukocytes and then pass into the vascular tissue independent of neutrophil extravasation. MPO is rapidly delivered to the endothelial cell via transcytosis, accumulates in the subendothelial zone, and exerts its effect in the vessel wall [25]. Kinetic studies have shown that MPO acts as a solvent pool for NO. It was shown that MPO decreases NO-dependent vasodilatation in isolated arteries and reduces NO bioavailability in cell cultures [28]. In our study, serum MPO levels at 24 h and 10 days were significantly higher in the IR group, preconditioning group, and Botulinum A toxin groups when compared with the control group. This indicates systemic neutrophil activation in these groups. The fact that this increase was more prominent in the preconditioning and Botulinum A toxin groups, illustrates the lack of correlation between tissue and serum MPO levels. Although the preconditioning method is reported to reduce the release of activated neutrophils [29], the high serum MPO levels in our study indicate the presence of activated leukocytes in the systemic circulation. The effect of Botulinum A toxin on neutrophils remains unknown. Considering that NO reduces leukocyte-neutrophil adhesion [30], preconditioning and Botulinum A toxin injection may reduce neutrophil adhesion by increasing NO, thus preventing neutrophil infiltration into the tissue. This may be interpreted as another mechanism preventing MPO transcytotic transport to endothelial tissue. Alternatively, MPO may be eliminated somehow by being released from tissue into the blood. It is not currently possible to state whether it is a local effect or a systemic one. MPO levels in the preconditioning group were markedly lower on day 10 compared to those at 24 h. This shows that although endothelial dysfunction and systemic neutrophil activity were somewhat decreased, the statistical nonsignificance of this reduction compared to the IR and Botulinum A toxin group indicates that neutrophil activation and endothelial dysfunction measured at 24 h persisted at nearly the same level on day 10 in these two groups. 

When all these data are taken into account, ischemic preconditioning and Botulinum A toxin have similar biochemical effects in the prevention of IRI on days 1 and 10. Our finding of lower MPO levels in the Botulinum A toxin group compared to the preconditioning group at 10 days may show that the Botulinum A toxin continued to protect the tissue from neutrophil-mediated damage on day 10, while the relatively lower tissue NO level in the Botulinum A group may indicate a reduced protective effect of NO or reestablishment of oxidant-antioxidant balance.

Because serum MPO and NO levels have not been used previously to evaluate IRI, our analysis of these values was based on intergroup comparisons and interpretation. Additional studies are needed for sounder analyses. As the present study was limited to 10 days, it precludes a full evaluation of tissue damage and highlights the need for longer-term studies. Although it has been stated that the duration of primary ischemia in musculocutaneous flaps is 9 h and secondary critical ischemia duration is 11 h [14], ischemia has been applied to musculocutaneous flaps for 4 h in experimental IR studies and resulted in macroscopic necrosis [31]. While it is emphasized in the literature that the presence of live skin tissue in flaps subjected to extended ischemia causes loss of muscle tissue viability [14], we did not observe gross necrosis in the present model in the IR-only group. Therefore, the use of an alternative model may be suggested for long-term studies.

Park et al. reported that Botulinum A toxin had a significant protective effect against IRI in a musculocutaneous flap model [32]. In our study, we compared Botulinum A toxin injection with the ischemic preconditioning procedure and determined that Botulinum A toxin protected against the IRI via increased NO and decreased MPO levels in the muscle tissue. According to tissue NO levels, preconditioning was superior to Botulinum A toxin, but we are unable to explain the high serum NO levels in the Botulinum A toxin group. Macroscopically, the flaps treated with Botulinum A toxin showed less contraction than flaps in the other groups (Figures 4 – 6). Çelik et al. showed previously that Botulinum A toxin injection is a useful method for stabilizing pedicled muscle flaps [4]. These effects could be considered an advantage of Botulinum A toxin.

In conclusion, Botulinum A toxin shows a protective effect against IRI in musculocutaneous flaps. Tissue MPO levels at 24 h suggest that ischemic preconditioning and Botulinum A toxin injection have equal protective effect, whereas Botulinum A toxin injection is superior to ischemic preconditioning at day 10. However, tissue NO levels indicate that ischemic preconditioning offers superior protection against IRI at both 24 h and 10 days.

**Conflict of interest**

The authors declare that they have no conflict of interest.
